# Lipidomic signatures in patients with early-onset and late-onset Preeclampsia

**DOI:** 10.1007/s11306-024-02134-x

**Published:** 2024-06-16

**Authors:** Yu Huang, Qiaoqiao Sun, Beibei Zhou, Yiqun Peng, Jingyun Li, Chunyan Li, Qing Xia, Li Meng, Chunjian Shan, Wei Long

**Affiliations:** 1 Department of Obstetrics, Women’s Hospital of Nanjing Medical University, Nanjing Women and Children’s Healthcare Hospital, 123rd Tianfei Street, Mochou Road, Nanjing, 210004 China; 2 Nanjing Maternal and Child Health Institute, Women’s Hospital of Nanjing Medical University, Nanjing Women and Children’s Healthcare Hospital, Nanjing, China; 3 Department of Obstetrics, Women’s Hospital of Nanjing Medical University, Nanjing Women and Children’s Healthcare Hospital, Nanjing, China

**Keywords:** Preeclampsia, Early-onset preeclampsia, Late-onset preeclampsia, Lipidomics, Biomarkers

## Abstract

**Background:**

Preeclampsia is a pregnancy-specific clinical syndrome and can be subdivided into early-onset preeclampsia (EOPE) and late-onset preeclampsia (LOPE) according to the gestational age of delivery. Patients with preeclampsia have aberrant lipid metabolism. This study aims to compare serum lipid profiles of normal pregnant women with EOPE or LOPE and screening potential biomarkers to diagnose EOPE or LOPE.

**Methods:**

Twenty normal pregnant controls (NC), 19 EOPE, and 19 LOPE were recruited in this study. Untargeted lipidomics based on ultra-performance liquid chromatography-tandem mass spectrometry (UPLC-MS/MS) was used to compare their serum lipid profiles.

**Results:**

The lipid metabolism profiles significantly differ among the NC, EOPE, and LOPE. Compared to the NC, there were 256 and 275 distinct lipids in the EOPE and LOPE, respectively. Furthermore, there were 42 different lipids between the LOPE and EOPE, of which eight were significantly associated with fetal birth weight and maternal urine protein. The five lipids that both differed in the EOPE and LOPE were DGTS (16:3/16:3), LPC (20:3), LPC (22:6), LPE (22:6), PC (18:5e/4:0), and a combination of them were a potential biomarker for predicting EOPE or LOPE. The receiver operating characteristic analysis revealed that the diagnostic power of the combination for distinguishing the EOPE from the NC and for distinguishing the LOPE from the NC can reach 1.000 and 0.992, respectively. The association between the lipid modules and clinical characteristics of EOPE and LOPE was investigated by the weighted gene co-expression network analysis (WGCNA). The results demonstrated that the main different metabolism pathway between the EOPE and LOPE was enriched in glycerophospholipid metabolism.

**Conclusions:**

Lipid metabolism disorders may be a potential mechanism of the pathogenesis of preeclampsia. Lipid metabolites have the potential to serve as biomarkers in patients with EOPE or LOPE. Furthermore, lipid metabolites correlate with clinical severity indicators for patients with EOPE and LOPE, including fetal birth weight and maternal urine protein levels.

**Supplementary Information:**

The online version contains supplementary material available at 10.1007/s11306-024-02134-x.

## Background

Pre-eclampsia is a pregnancy-specific clinical syndrome characterized primarily by new-onset hypertension and proteinuria after 20 weeks of pregnancy, with a worldwide incidence of approximately 2–8% (Ives et al., 2020). Preeclampsia increases the risk of adverse events for both the mother and the fetus, such as maternal kidney injury, liver damage, hematopoietic and neurological system complications, and abnormal fetal-maternal placental circulation (fetal growth restriction, placental abruption, premature birth, fetal death, etc.) (Erez et al., 2022; Jena et al., 2020). The primary clinical management of preeclampsia is using oral labetalol, methyldopa, and magnesium sulfate to control maternal blood pressure for slowing disease progression (Amaral et al., 2017). However, the only effective treatment for preeclampsia is early delivery of the fetus and the placenta; this often results in preterm birth and low birth weight infants (Beckers et al. 2020).

The precise pathogenesis of preeclampsia is unclear; it is currently known possible pathogenic mechanisms include insufficient trophoblast invasion and uterine spiral artery remodeling disorder, placental hypoxia, and release of circulating anti-angiogenic factors, and immune imbalance at the maternal-fetal interface (Ives et al., 2020). According to the gestational age of delivery, preeclampsia can be divided into two subtypes: early-onset preeclampsia (EOPE, delivery at < 34 + 0 weeks of gestation) and late-onset preeclampsia (LOPE, delivery at ≥ 34 + 0 weeks of gestation) (Poon et al., 2019a). Studies have discovered that EOPE and LOPE exhibit different pathogenesis, risk factors, clinical features, biomarkers, and maternal-fetal outcomes. Therefore, they should be considered as two distinct disease entities (Raymond et al. 2011; Roberts et al., 2021). It is widely accepted that EOPE is primarily recognized as a placenta-derived disease triggered by dysfunctional placental trophoblast cells. In contrast, LOPE is commonly regarded as a metabolic syndrome resulting from fetal-maternal metabolic imbalance (Ren et al., 2021; Staff, 2019). Patients with EOPE have more serious clinical manifestations than those with LOPE, including elevated blood pressure and urine protein levels, higher rates of perinatal mortality, and a greater incidence of severe neonatal morbidity (Lisonkova et al. 2013). Furthermore, when compared to normal pregnant women, the alterations in biomarkers such as soluble fms-like tyrosine kinase 1 (sFlt-1), leptin, fibronectin, and placental exosomes were more pronounced in EOPE than those in LOPE (Masuyama et al., 2010).

Previous studies have identified some biomarkers for the prediction of preeclampsia, such as sFlt-1, sFlt-1/placental growth factor (PlGF), mean arterial pressure (MAP), uterine artery pulse index (UtA-PI), and pregnancy-associated plasma protein A (PAPP-A) (Ansbacher-Feldman et al., 2022; Zeisler et al., 2016). Moreover, recent studies have reported that maternal plasma cell-free DNA, acute-phase protein response to inflammation, and circulating hypertension-associated peptides may be potential diagnostic biomarkers for preeclampsia (Bokuda et al. 2023). However, these biomarkers cannot distinguish between and predict two different subtypes of preeclampsia, namely EOPE and LOPE. Therefore, developing novel prediction tools to identify different subtypes of preeclampsia is urgently necessary.

Lipids are ubiquitous in living organisms, constitute the fundamental composition of cell membranes, participate in various physiological processes within the body, and are essential for cell signaling and energy metabolism (Kimura et al., 2016). As a branch of metabolomics, lipidomics can reflect the physiological and pathological conditions in living organisms and reveal the key role of lipids in cellular function and disease mechanisms (Kvasnička et al., 2023). Lipids play an important role in pregnancy-related diseases, such as gestational diabetes, fetal growth restriction, and recurrent miscarriage. Lipidomics can provide novel biomarkers for these diseases and clarify the metabolic pathways involved in disease onset and progression, opening up new prospects for a comprehensive understanding of disease pathophysiology(Canella et al., 2023; Miranda et al., 2018; Wang et al., 2023b). Several studies have demonstrated that lipid metabolism disorders contribute to the development of preeclampsia (Enquobahrie et al., 2004; Negre-Salvayre et al., 2022). Patients with preeclampsia have aberrant lipid metabolism, manifested as hyperlipidemia, increased total cholesterol, triglycerides, free fatty acids, low-density lipoprotein, and decreased high-density lipoprotein (Spracklen et al. 2014; Villa et al. 2009). Elevated levels of lipid peroxides contribute to heightened oxidative stress and inflammation inside the body, leading to harm to vascular endothelial cells. Moreover, the accumulation of lipids in the spiral artery of preeclampsia patients induces sudden atherosclerotic alterations and exacerbates placental ischemia and hypoxia, hence facilitating the development of preeclampsia (Staff et al., 2013). As a result, lipidomics research on preeclampsia can thoroughly and methodically elucidate the role of lipids in preeclampsia pathophysiology, offering novel perspectives on pathogenesis, biomarker identification, and personalized treatment of preeclampsia. However, there is a dearth of research on the relevance of different lipidomic profiles between EOPE and LOPE. Given the differences in pathogenesis, clinical presentations, and prognosis between EOPE and LOPE, studies of their unique lipid metabolic profiles could facilitate the identification of their respective biomarkers and provide innovative perspectives on their clinical management.

Therefore, this study utilizes UPLC-MS/MS to identify serum differential lipids profiles among patients with EOPE and LOPE and healthy pregnant women, find differential characteristic lipid metabolites and evaluate their diagnostic efficacy as potential biomarkers, aid in the timely diagnosis of preeclampsia, and increase understanding of the pathogenesis of preeclampsia from a lipidomics perspective.

## Methods

### Study participants

The acquisition of participant serum samples was approved by the Ethics Committee of the Women’s Hospital of Nanjing Medical University (License Number. 2023KY-086). All participants supplied their signed informed consent and agreed to peripheral blood collection. The pregnant women in the normal control groups had normal blood pressure during pregnancy and were full-term singleton pregnancies without any complications. preeclampsia is diagnosed according to the International Federation of Gynecology and Obstetrics (FIGO) guidelines (Poon et al., 2019b). Preeclampsia is characterized as new-onset hypertension (blood pressure greater than 140/90 mmHg) after 20 weeks of gestation, along with proteinuria (≥ 0.3 g/24 hours) or in the absence of proteinuria, new-onset hypertension accompanied by any of other new symptoms: renal insufficiency, impaired liver function, thrombocytopenia, pulmonary edema, new-onset headache unresponsive to medication or visual symptoms. EOPE is defined as delivery < 34 weeks gestation, and LOPE is defined as delivery ≥ 34 weeks. Exclusion criteria were multiple pregnancies, endocrine-gynecology disorders (e.g., polycystic ovary syndrome and endometriosis), chronic hypertension, pre-existing diabetes, chronic liver or kidney disease, thyroid insufficiency, cardiovascular disease, autoimmune disease (e.g., antiphospholipid syndrome, systemic lupus erythematosus), and infectious disease (e.g., sexually transmitted disease, chorioamnionitis, urinary tract infection). All the participants were matched for maternal age.

From May 2022 to January 2023, 20 healthy pregnant women (normal control group, NC), 19 women with EOPE, and 19 women with LOPE were recruited and sampled prior to delivery. All participants fasted overnight, and fasting blood samples were collected the following morning. After sitting for 1 h at room temperature, blood samples were centrifuged at 12,000 *rpm* for 10 min at 4 °C. The serum supernatants were collected and stored at -80 °C for later analysis.

### Lipid extraction

First, 100 µL of the serum sample was added to 480 µL of extract solution (MTBE: MeOH = 5:1). The mixture was vortexed for 30 s, sonicated in an ice water bath for 10 min, and then incubated at -40 °C for 1 h. The samples were centrifuged at 3000 *rpm* for 15 min at 4 °C, and 350 µL of the supernatant was collected and dried under vacuum. Then 100 µL of the solution (DCM: MeOH = 1:1) was added to redissolve, vortexed for 30 s, and sonicated in an ice water bath for 10 min. The samples were centrifuged at 12,000 *rpm* for 15 min at 4 °C. Finally, 90 µL of supernatant was transferred to the injection container for LC-MS detection.

### Untargeted lipidomic analysis

To ensure the stability of the UHPLC-QE-MS system, an additional 10 µL of residue was mixed into quality control (QC) samples for machine testing. The untargeted LC-MS/MS detection system employs the Vanquish (Thermo Fisher Scientific) ultra-performance liquid chromatograph with Phenomen Kinetex C18 (2.1*100 mm, 1.7 m) liquid chromatographic column. The column temperature was 55 °C, mobile phase A consisted of water and acetonitrile solution (volume 4:6, containing 10 mmol/L ammonium formate), mobile phase B was composed of acetonitrile and isopropanol (volume 9:1, containing 50 mL of 10 mmol/L ammonium formate per 1000 mL). The flow rate was 0.3 mL/min, the sample dish temperature was 4 °C, and the injection volume was 2 µL of positive and negative ions 2 µL. The elution gradient is as follows: ~1 min, 40% mobile phase B; 1 ~ 12 min, 40%~100% mobile phase B; 12 ~ 13.5 min, 100% mobile phase B; 13.5 ~ 13.7 min, 40%~100% mobile phase B; 13.7 ~ 18.0 min, 40% mobile phase B.

Following that, a Thermo Q Exactive Orbitrap mass spectrometer was used to obtain mass spectrum data under the supervision of control software (Xcalibur, version 4.0.27, Thermo). Detailed parameters are sheath gas flow rate: 30 arb, aux gas flow rate: 10 arb, capillary temperature: 320 °C (positive) or 300 °C (negative), full MS resolution: 70,000, MS/MS resolution: 17,500, collision energy: 15/30/45 in NCE mode, spray voltage: 5 kV (positive) or -4.5 kV (negative). All sample analysis techniques use the same MS scanning parameters.

### Raw data processing

The original raw lipidomics data obtained by the mass spectrometry analysis software was converted into mzXML format by ProteoWizard software and then processed using an internal program developed by R software, which was preprocessed based on XCMS for retention time correction, peak identification, peak filtration, peak extraction, peak alignment, etc., with the set parameters as follows: minfrac set to 0.5, cutoff set to 0.3, and lipid identification were performed using the lipidblast database.

### Statistical analysis

SIMCA software 16.0.2 (Sartorius Stedim Data Analytics AB, Umea, Sweden) was used for principal component analysis (PCA) and orthogonal partial least squares discriminant analysis (OPLS-DA). PCA was unsupervised, which was used to explore overall serum lipid profile differences between groups. The OPLS-DA model is based on a supervised grouping pattern to eliminate the influence of irrelevant factors in the experiment. Each lipid has a variable importance in the project (VIP) value which is used to determine the contribution of the lipid in the differentiation between groups and evaluate the model with R²Y and Q². The larger R²Y and Q² values indicate the model has great predictive efficiency and explanatory ability. Lipids with significant differences were selected based on the VIP value > 1 and *p-value* < 0.05 compared between groups.

Clinical data were statistically analyzed using SPSS 26.0 (IBM, Armonk, NY, USA) software. The Chi-Square, Kruskal-Wallis H tests, Mann-Whitney U, analysis of variance (ANOVA) test, and Fisher exact tests were used for intergroup comparison. Data are shown as mean ± standard deviation. R software (version 4.2.0) was used to draw volcano and bubble plots. MetaboAnalyst software (version 5.0) and the KEGG database were used to analyze the metabolic pathways of lipids with significant differences between groups. The relationship between serum lipids of EOPE and LOPE and their clinical characteristics was analyzed with the weighted lipid co-expression network analysis based on the R package “WGCNA” (Langfelder et al. 2008). IBM SPSS Statistics (version 26.0) for Spearman correlation analysis. GraphPad Prism (version 9.5.1) for correlation scatter plots. The receiver operating characteristic curve (ROC) was plotted by the MedCalc software (version 20.022) to assess the diagnostic capability of these candidate lipids, the area under the curve (AUC) values reflecting diagnostic power.

## Results

### Baseline characteristics of the participants

This study involved 58 participants, including 20 normal pregnant women in the NC group, 19 patients with EOPE, and 19 patients with LOPE. Table 1 lists the detailed clinical characteristics of the subjects. There were no significant differences in maternal age, proportion of IVF, or platelet count. The EOPE or LOPE groups had significantly higher levels of maternal BMI, SBP, DBP, blood BNP concentration, 24-hour proteinuria, ALT, and AST than the NC group (*p* < 0.05). The EOPE group had significantly higher levels of blood BNP concentration and 24-hour proteinuria than the LOPE group (*p* < 0.05). But we found no significant differences in maternal BMI, SBP, DBP, ALT, and AST levels between the EOPE and LOPE groups. In addition, the mean GA at delivery and the fetal birth weight in the EOPE group were lower than those in the LOPE group or the NC group (*p* < 0.001), but there was no significant difference between the LOPE and the NC groups.

It can be seen that the EOPE group had more severe clinical features than the LOPE group, such as maternal urine protein levels being significantly higher in the EOPE group than in the LOPE group (5.26 ± 2.91 g/24 h vs. 0.56 ± 0.61 g/24h, *p* < 0.001), while fetal birth weight was significantly lower than that in the LOPE group (1736.00 ± 469.21 g vs. 2885.26 ± 650.10 g, *p* < 0.001).

**Table 1:** Baseline characteristics of the participants

Characteristics	NC (*n* = 20)	EOPE (*n* = 19)	LOPE (*n* = 19)	*p*-value	LOPE vs. EOPE	EOPE vs. NC	LOPE vs. NC
**Maternal age (years)**	30.15 ± 3.54	30.68 ± 3.61	29.42 ± 3.66	0.439	—	—	—
**BMI (Kg/m²)**	25.31 ± 2.13	28.13 ± 3.31	30.74 ± 3.76	**< 0.001**	0.187	**0.014**	**< 0.001**
**SBP (mmHg)**	115.70 ± 8.86	144.00 ± 15.80	139.79 ± 11.75	**< 0.001**	1.000	**< 0.001**	**< 0.001**
**DBP (mmHg)**	72.40 ± 4.33	96.22 ± 9.44	88.68 ± 8.05	**< 0.001**	0.336	**< 0.001**	**< 0.001**
**GA at delivery (weeks)**	39.01 ± 1.76	31.53 ± 4.12	38.21 ± 1.43	**< 0.001**	**< 0.001**	**< 0.001**	1.000
**Fetal birth weight (g)**	3103.00 ± 445.76	1736.00 ± 469.21	2885.26 ± 650.10	**< 0.001**	**< 0.001**	**< 0.001**	0.847
**IVF (%)**	0	4 (21.1)	2 (10.5)	0.072	0.660	**0.047**	0.231
**Proteinuria (g/24 h)**	—	5.26 ± 2.91	0.56 ± 0.61	**< 0.001**	**< 0.001**	—	—
PLT (*10^9/L)	209.20 ± 51.70	180.47 ± 66.31	218.74 ± 76.23	0.180	0.076	0.175	0.650
**BNP (pg/mL)**	—	342.47 ± 335.62	81.56 ± 98.65	**0.012**	**0.012**	—	—
**ALT (U/L)**	10.82 ± 5.33	22.95 ± 12.54	19.66 ± 10.50	**< 0.001**	1.000	0.001	**< 0.001**
**AST (U/L)**	17.12 ± 3.73	26.92 ± 9.94	24.63 ± 7.84	**< 0.001**	1.000	**< 0.001**	**0.001**

Data are presented as mean ± SD or n (%). BMI, body mass index; SBP, systolic blood pressure; DBP, diastolic blood pressure; GA, gestational age; IVF, in vitro fertilization; PLT, platelet; BNP, brain natriuretic peptide; ALT, alanine transaminase; AST, aspartate aminotransferase

### Separation of serum lipid profiling among the NC, EOPE, and LOPE groups

**Fig. 1:**
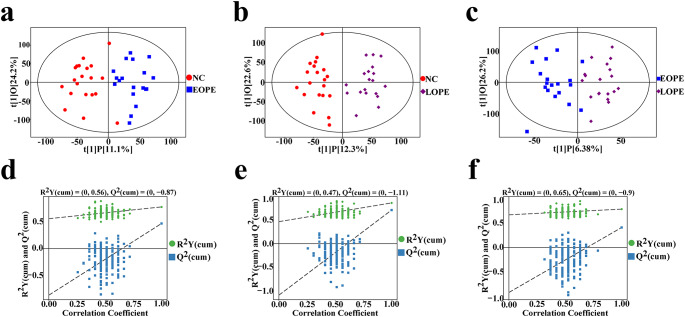
LC-MS/MS lipidomics analysis of NC, EOPE, and LOPE. **a ~ c**. The OPLS-DA model of the NC, EOPE and LOPE groups. The red dots represent NC, the blue dots represent EOPE, and the purple dots represent LOPE. **d ~ f.** Scatter plot of OPLS-DA model and validation model of permutation test between two groups. **a, d.** NC vs. EOPE. **b, e.** NC vs. LOPE **c, f.** EOPE vs. LOPE. OPLS-DA, orthogonal partial least square discriminant analysis; NC, normal control group; EOPE, early-onset preeclampsia; LOPE, late-onset preeclampsia

UHPLC-QE-MS untargeted lipidomics was used to compare the alterations in serum lipids between the EOPE, LOPE and NC group. QC sample monitoring instrument fluctuations were employed during the analysis to ensure the collected data’s quality. The TIC of the QC sample was superimposed in the positive and negative ion modes. The retention time and peak area of TIC are well overlapped, indicating the strong stability and repeatability of the system (Figure S1). After normalization with QC data and raw data processing, 10,107 peaks in the positive and 8191 peaks in the negative ion modes were maintained (Table S1). Matching with the LipidBlast database, 1036 lipids were identified according to their MS2 fragmentation information. The OPLS-DA model based on multivariate supervision was employed to compare the lipid profiles between the EOPE and the NC group. The OPLS-DA model scatter plot revealed a significant separation between the EOPE and the NC group (Fig. 1a). The permutation test of the model showed that the R2Y was 0.56 and Q2 was 0.87 (Fig. 1d). Similarly, the separations were observed in lipid profiles between the LOPE and the NC group (Fig. 1b**).** The permutation test of the model showed that the R2Y was 0.47 and Q2 was 1.11 (Fig. 1e). The EOPE and the LOPE group can also be distinguished by OPLS-DA model (Fig. 1c**).** The permutation test of the model showed that the R2Y was 0.65 and Q2 was 0.90 (Fig. 1f). Therefore, these OPLS-DA models were not over-fitting and dependable for further screening potential lipid biomarkers.

### The lipid species that exhibited significant differences among the NC, EOPE, and LOPE group

**Fig. 2:**
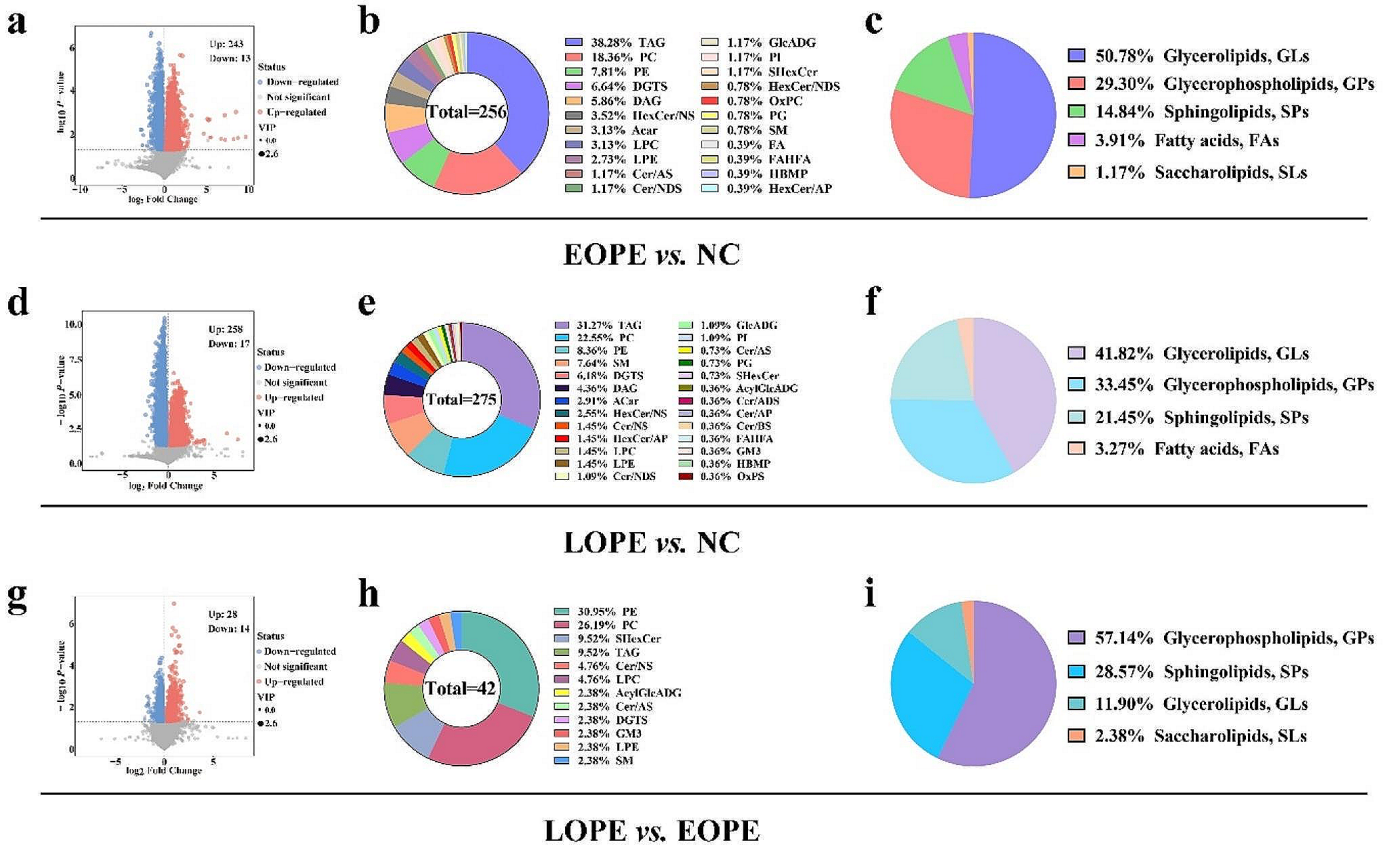
Serum lipid species differed among the NC, EOPE, and LOPE groups. The volcano plots **(a)**, pie charts **(b)**, and **(c)** show differential lipids, differential lipid species composition, and corresponding subclasses between the EOPE and the NC groups, respectively. The volcano plots **(d)**, pie charts **(e)**, and **(f)** show differential lipids, differential lipid species composition, and corresponding subclasses between the LOPE and the NC groups, respectively. The volcano plots **(g)**, pie charts **(h)**, and **(i)** show differential lipids, differential lipid species composition, and corresponding subclasses between the LOPE and the EOPE groups, respectively

Based on the OPLS-DA model, differential lipids between the two groups were screened according to VIP > 1, *p-value* < 0.05. The volcano plots visualized significant variances in lipids between the two groups. The size of the dots represents VIP values, red dots represent up-regulated lipids, blue dots represent down-regulated lipids, and gray dots show insignificant changes. In comparison to the NC group, there were 275 lipids that were significantly different (including 258 up-regulated and 17 down-regulated) in the EOPE group (Fig. 2a). Similarly, there were 256 differential lipids in the LOPE group (243 up-regulated and 13 down-regulated) (Fig. 2d). A total of 42 lipids significantly differed between the LOPE and the EOPE groups (including 28 up-regulated and 14 down-regulated) (Fig. 2g). We performed pie chart analysis on the class of differential lipids between the NC, EOPE, and LOPE groups and further analyzed the composition of the differential lipid species and corresponding subclasses between the groups. The lipid species that showed the most significant differences between the NC group and the EOPE or LOPE groups were triacylglycerol (TAG), phosphatidylcholine (PC), and phosphatidylethanolamine (PE) (Fig. 2b, e). The lipid species that exhibited substantial differences between the EOPE and LOPE groups were PE, PC, and sulfide hexoceramide (SHexCer) (Fig. 2h). Furthermore, the lipid subclasses with the most significant changes between the EOPE group and the NC group were 130 glycerolipids (GLs), 75 glycerophospholipids (GPs), 38 sphingolipids (SPs), 10 fatty acids (FAs), and 3 glycolipids (SLs) (Fig. 2c). The lipid subclasses that changed most significantly between the LOPE group and the NC group included 115 GLs, 92 GPs, 59 SPs, and 9 FAs (Fig. 2f). Compared with the LOPE group and the EOPE group, the lipid subclasses with the most significant changes included 24 GPs, 12 SPs, 5 GLs, and 1 SL (Fig. 2i).

**Fig. 3:**
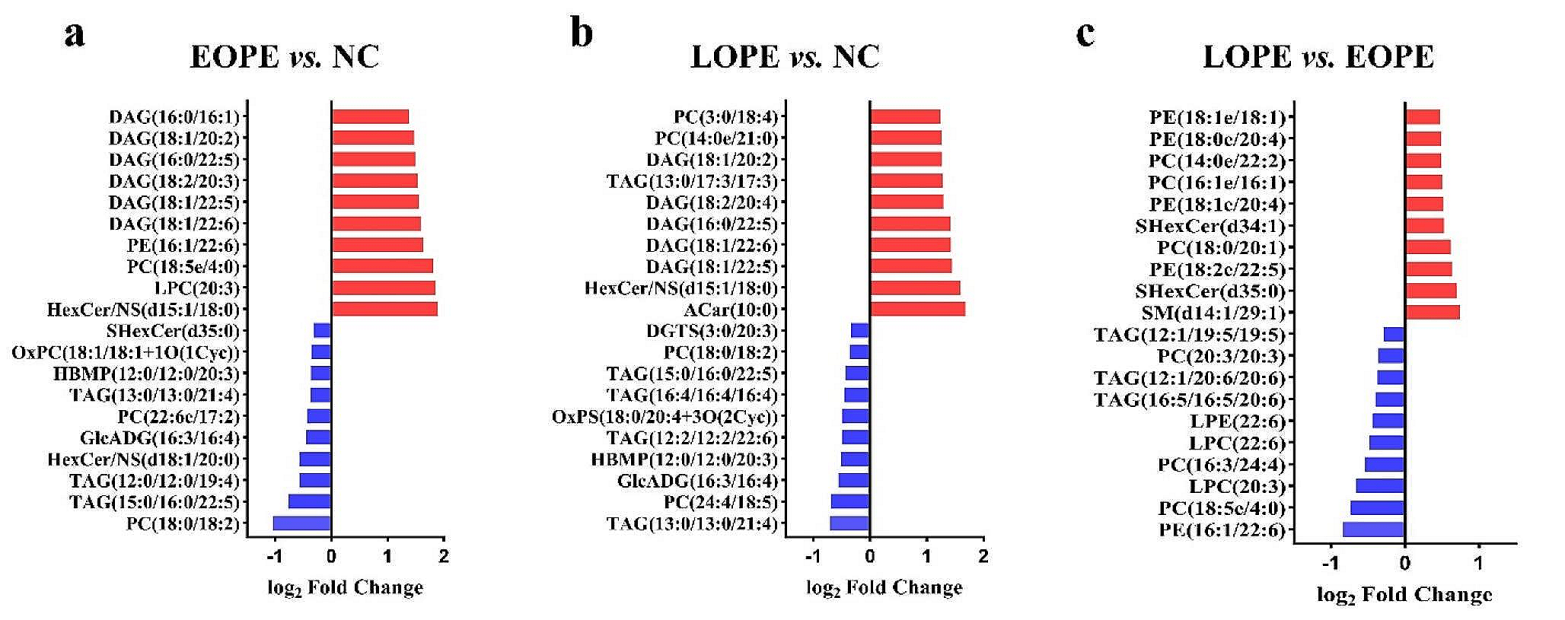
The matchstick plots reveal the top 20 lipids significantly up-regulated and down-regulated among the NC, EOPE, and LOPE groups **(a)** The top 20 significantly changed lipids between the NC group and the EOPE group. **(b)** The top 20 significantly changed lipids between the NC group and the LOPE group. **(c)** The top 20 significantly changed lipids between the EOPE group and the LOPE group

Furthermore, HexCer/NS(d15:1/18:0), LPC(20:3), and PC(18:5e/4:0) were the most significantly changed lipids between the NC and the EOPE group. When comparing the LOPE and the NC group, the lipids that significantly changed were ACar(10:0), HexCer/NS(d15:1/18:0), and DAG(18:1/22:5). When comparing the EOPE and the LOPE group, the most significantly changed lipids were PE(16:1/22:6), SM(d14:1/29:1) and PC(18:5e/4:0). The top 20 significantly up-regulated and down-regulated lipids in the NC and EOPE, the NC and LOPE, and the EOPE and LOPE groups were respectively shown in Fig. 3a **~ c**.

### Clinical characteristics of EOPE and LOPE correlated to their distinct lipid network modules

**Fig. 4:**
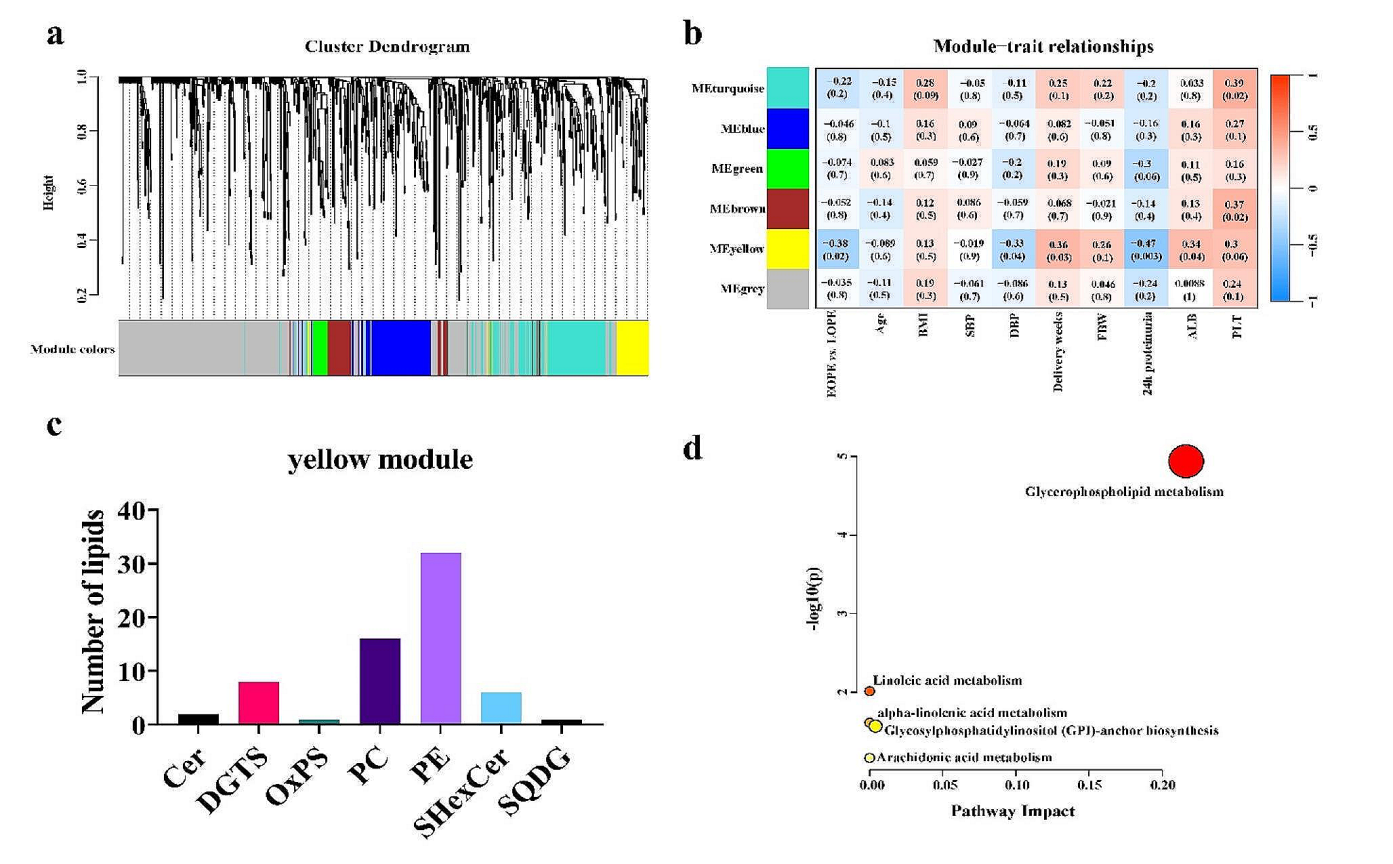
Clinical characteristics of the EOPE and the LOPE group correlate to distinct lipid network modules. **(a)** Module clustering trees were used to visualize the distribution of lipids of the EOPE group and the LOPE group in each module. **(b)** The correlation coefficient and *p-value* of lipid modules with the clinical characteristics of the EOPE and the LOPE groups. **(c)** The number of lipid metabolites species in the yellow module. **(d)** Metabolic pathways analysis of hub lipids

To explore the serum lipid clusters highly correlated with clinical characteristics of EOPE and LOPE, we performed WGCNA analysis to construct the co-expression network among all identified lipids for EOPE and LOPE (Langfelder, & Horvath, 2008). First, the hierarchical clustering algorithm was performed to determine the outliers among the serum lipid profile samples, and no abnormal samples were detected (Fig. S2). Second, the scale-free network was used to select the optimal soft-threshold power, and a topological overlap matrix (TOM) was constructed (Fig. S2). Then, based on the scale-free network, the optimal soft threshold power of 14 was selected, the mergeCutHeight was set to 0.25, and the minModuleSize was 30. The 1036 identified lipids with highly similar expression matrices were divided into different modules based on their internal connectivity, and the cluster dendrogram plot was used to visualize it (Fig. 4a).

Finally, a total of 6 modules were obtained. The turquoise, blue, green, brown, yellow, and grey modules contain 204, 137, 35, 76, 66, and 518 lipids, respectively (Table S2). The grey modules consist of lipids not grouped into any co-expression module. Module-trait relationships plot revealed that the yellow module was highly correlated with the diastolic blood pressure (*r*=-0.33, *p* = 0.04), delivery weeks (*r* = 0.36, *p* = 0.03), 24 h proteinuria (*r*=-0.47, *p* = 0.003) and ALB (*r* = 0.34, *p* = 0.04) of the EOPE and the LOPE group (Fig. 4b), and it mainly contains PEs and PCs (Fig. 4c). To explore the different metabolism pathways between the EOPE and LOPE. We performed the pathway analysis of lipids in the yellow module based on the MetaboAnalyst platform. The results revealed that the main pathway is enriched in glycerophospholipid metabolism (Fig. 4d).

### Screening the potential lipid biomarkers for EOPE and LOPE diagnosis

**Fig. 5:**
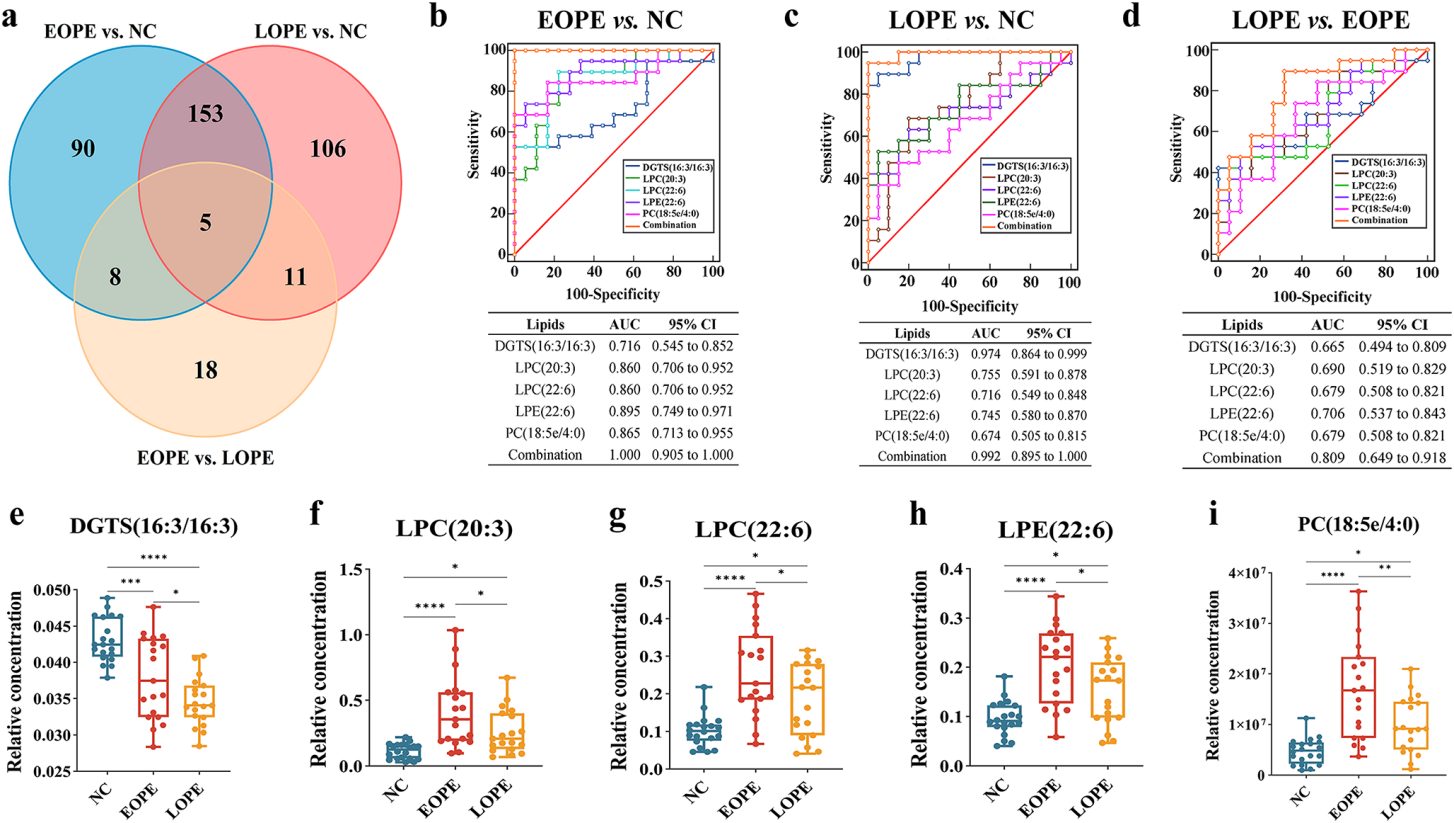
Screening the potential lipid biomarkers for EOPE and LOPE diagnosis. **(a)** Venn plot depicts significant lipid numbers among the NC, EOPE, and LOPE groups. **(b)** Five different lipids distinguish the EOPE group from the NC group. **(c)** Five different lipids distinguish the LOPE group and the NC group. **(d)** Five different lipids distinguish the LOPE group and the EOPE group. **e-i.** The relative concentration of DGTS (16:3/16:3), LPC (20:3), LPC (22:6), LPE (22:6), and PC (18:5e/4:0) in the NC, EOPE, and LOPE groups, respectively

The Venn diagram showed the number of lipids significantly expressed differently among the three groups (Fig. 5a). ANOVA test results showed that the five lipids presented significant statistical differences among the three groups were DGTS(16:3/16:3), LPC(20:3), LPC(22:6), LPE(22:6) and PC(18:5e/4:0). Considering that there was a significant difference in maternal BMI between the NC group and the EOPE or LOPE groups, we performed covariance analysis to account for the potential impact of BMI on the maternal serum lipid profile in this study. Our analysis revealed that the BMI had no significant impact on the expression of the five lipids that were shown to be significantly different among the three groups. The relative concentration levels of DGTS(16:3/16:3) were significantly reduced in EOPE or LOPE groups compared to the NC group, and this reduction was more pronounced in the LOPE group than in the EOPE group (Fig. 5e**)**. The relative concentration levels of LPC(20:3), LPC(22:6), LPE(22:6), and PC(18:5e/4:0) were significantly higher in the EOPE or LOPE group compared to the NC group, and the EOPE group exhibited higher levels than the LOPE group (Fig. 5f **~ i**). The ROC curves were used to analyze the diagnostic efficacy of 5 lipids, which were significantly expressed differently among EOPE vs. NC, LOPE vs. NC, and LOPE vs. EOPE. The panel of five differential lipids can robustly distinguish the patients with EOPE from the NC group, with the largest area under the ROC curve (AUC) was 1.000 (95%CI: 0.905 to 1.000) (Fig. 5b). When distinguishing the patients with LOPE from the NC group, the area under the ROC curve (AUC) was 0.992 (95%CI: 0.895 to 1.000) (Fig. 5c), as well as distinguish the LOPE group from the EOPE group, the area under the ROC curve (AUC) was 0.809 (95%CI: 0.649 to 0.918) (Fig. 5d). LPE showed good diagnostic efficacy in distinguishing the EOPE from the NC group (AUC = 0.865, 95% CI (0.749–0.971)) and the LOPE from the EOPE group (AUC = 0.706, 95% CI (0.537–0.843)), respectively. DGTS demonstrated great diagnostic efficacy in distinguishing the LOPE from the NC group (AUC = 0.974, 95% CI (0.864 to 0.999)).

### Maternal specific serum lipids associated with proteinuria and fetal birth weight of EOPE and LOPE

In our study, EOPE patients had higher levels of BNP and maternal urine protein than LOPE patients, while fetal birth weight was significantly lower than LOPE patients. We conducted the Spearman correlation analysis on 42 lipid metabolites that exhibited differently between patients with EOPE and LOPE, including maternal clinical characteristics such as BNP, fetal birth weight (FBW), and 24-hour urine protein. The results were visualized using a correlation heat map (Fig. S3).

**Fig. 6:**
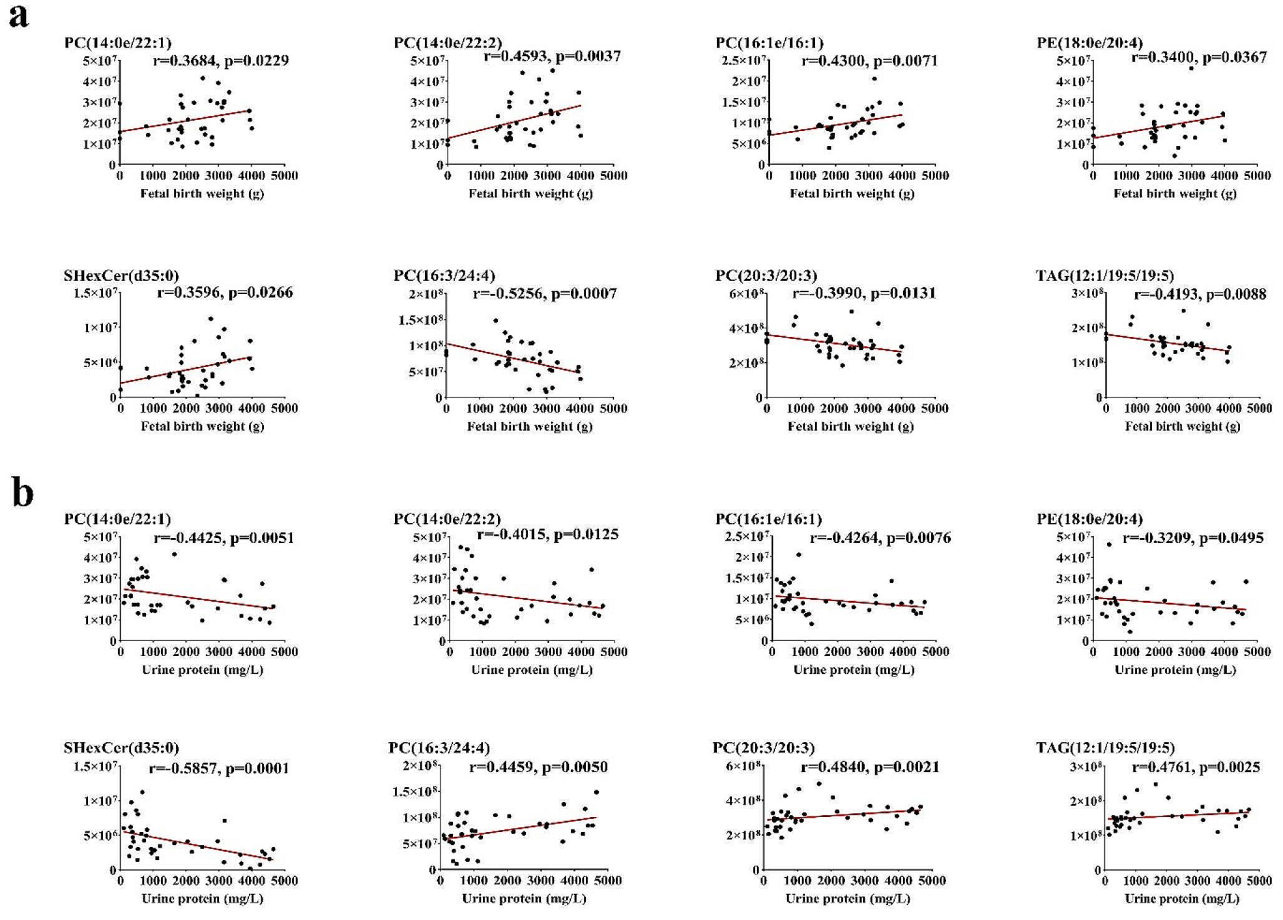
Correlations between specific differential maternal serum lipids and clinical parameters of patients in the EOPE and the LOPE groups. **(a)** Scatterplots depict the correlation between lipids and fetal birth weight. **(b)** Scatterplots show the correlation between lipids and maternal urine protein

The correlation analysis revealed a statistically significant association between 18 lipids and fetal birth weight, as well as a statistically significant association between 24 lipids and maternal urine protein levels (S3). Eight lipids were both significantly correlated with maternal urine protein levels (Fig. 6a**)** and fetal birth weight (Fig. 6b**)**, including PC (14:0e/22:1), PC (14:0e/22:2), PC (16:1e/16:1), PC (20:3/20:3), PC (16:0e/22:6), PE (18:0e/20:4), SHexCer (d34:1), and SHexCer (d35:0). Among the 8 lipids, PC(14:0e/22:1), PC(14:0e/22:2), PC(16:1e/16:1), PE(18:0e/20:4), and SHexCer(d35:0) were positively correlated with fetal birth weight while negatively correlated with maternal urinary protein. PC(16:3/24:4), PC(20:3/20:3), and TAG(12:1/19:5/19:5) were negatively correlated with fetal birth weight while positively correlated with maternal urinary protein.

## Discussion

Preeclampsia is a serious disease of pregnancy that adversely affects both the mother and the fetus. The treatment of preeclampsia is limited; once it develops, it can only be relieved of its threat to the mother and fetus by early labor induction. EOPE patients usually have more serious clinical symptoms and earlier delivery time than LOPE patients; it is more likely to cause adverse maternal and fetal outcomes. Therefore, more research is required to investigate the potential causes of the different subtypes of preeclampsia, especially EOPE, and offer new perspectives for its diagnosis and therapy.

In this study, we found that the lipid species with the most significant changes in preeclampsia compared with normal pregnant women were TAGs, PCs, and PEs, which was consistent with the conclusions of previous studies (Brown et al., 2016). Triglycerides contribute to the development of preeclampsia by affecting pregnant women’s BMI, causing damage to vascular endothelial cells, and triggering inflammatory responses (Bodnar et al., 2005). Amor et al. discovered a significant correlation between the level of PC in women with a history of preeclampsia and the presence of atherosclerosis. This suggests that the detrimental impact of PC on the blood vessels of preeclampsia patients may even persist into the postpartum period (Amor et al., 2021). Furthermore, Jääskeläinen et al. discovered a significant increase in PC levels in umbilical cord plasma in preeclampsia neonates (Jääskeläinen et al., 2018). PE is one of the most abundant phospholipids in living organisms and is involved in a variety of life processes, such as promoting oxidative phosphorylation, inducing lipid peroxidation, endoplasmic reticulum stress, and promoting ferroptosis (Lee et al., 2021). Injection of mixed phosphatidylserine (PS) / PC microvesicles into mice’s tail vein can induce preeclampsia-like changes such as intrauterine growth restriction, increased systolic blood pressure, and placental vascular hypercoagulation (Omatsu et al., 2005).

We found that there were differences in lipid metabolism in patients with EOPE and LOPE, mainly PEs, PCs, and ceramides. The WGCNA analysis showed that the clinical characteristics of patients with EOPE or LOPE were strongly associated with PEs and PCs. Additionally, the metabolic pathways with the most significant differences between EOPE and LOPE were mainly enriched in the glycerophospholipid metabolism pathway. Therefore, these two lipid species located in the glycerophospholipid metabolism pathway may be involved in the development of preeclampsia into EOPE or LOPE. Abnormal elevation of ceramide can increase transcription factor EB (TFEB) expression and nuclear translocation, induce lysosomal formation and exocytosis, and lead to excessive autophagy and programmed necrosis of trophoblasts, resulting in the onset of preeclampsia (Liao et al., 2022). In addition, a comparison of lipid subclasses in EOPE and LOPE compared to normal pregnant women suggests that SLs may play a role in the pathogenesis of EOPE. SLs are lipids formed by esterifying fatty acids linked to a glycosidium, which can act as receptors for bacteria or viruses and are involved in disease development (Wang et al., 2023a). Akyol et al. discovered that SLs play a crucial role in maintaining the integrity of the neuronal membrane structure and serve as the starting point for bioactive brain lipids. These lipids have a crucial impact on synaptic transmission and stabilization, neuronal survival, and cell signal transduction. However, when inflammatory factors activate and lose their myelin sheath, they can exacerbate the symptoms of brain diseases like Alzheimer’s (Akyol et al., 2021). Our study demonstrates that EOPE patients have aberrant alterations in SLs, potentially contributing to the heightened severity of clinical symptoms and increased susceptibility to neurological manifestations.

Furthermore, our study revealed that the LOPE group had a significantly higher maternal BMI than the NC group, but there was no significant difference in the fetal birth weight between the two groups. This may be attributed to the pathogenic mechanism of LOPE, which is often associated with the mother’s metabolic disorders, potentially causing less harm to the fetus. The fetal birth weight in the EOPE group was significantly lower than that in the NC group, which may be because EOPE was more manifested as placental abnormalities, causing insufficient blood supply and dysfunction of the placenta, leading to subsequent fetal growth restriction. Moreover, because the gestational age of admission in the LOPE patient group in our study was usually greater than 34 weeks, the harm to the mother and fetus was usually less than that of the EOPE group, and the clinical manifestations were milder than those of the EOPE patient group. Therefore, we did not take into account the possible effect on lipid profiles in patients with severe LOPE, and it may be possible to stratify EOPE and LOPE in subsequent studies according to clinical severity.

Recently, liquid chromatography-mass spectrometry has been widely employed in lipidomics research. This provides a deeper understanding of the pathogenesis of preeclampsia from the perspective of lipid metabolism and enables the identification of biomarkers that can enhance the prediction, diagnosis, and treatment of preeclampsia (Stephenson et al., 2017; Zhang et al., 2022). In a cohort study of maternal plasma samples collected at 36 weeks of gestation, Bartho et al. found that phosphatidylinositol 32:1 was effective in predicting LOPE after 36 weeks of gestation (AUC = 0.81) (Bartho et al., 2023). Therefore, it is possible to compare the differences in lipid profiles of patients with EOPE or LOPE to identify specific lipid biomarkers related to EOPE and LOPE and explore their underlying pathogenesis.

In this study, we performed an untargeted lipidomics analysis to detect serum from normal pregnant women and patients with EOPE and LOPE. In our study, we observed that the EOPE group had 256 different lipids compared to the NC group, while the LOPE group had 275 different lipids compared to the NC group. Additionally, there were 42 different lipids in the LOPE and EOPE groups. Out of the 42 different lipids, eight exhibited significant correlations with both fetal birth weight and maternal urine protein. Among these lipids, five displayed a positive correlation with fetal birth weight and a negative correlation with maternal urine protein levels, while three exhibited a negative correlation with fetal birth weight and a positive correlation with maternal urine protein levels. The five lipids that all showed significant differences in the NC, EOPE, and LOPE groups were DGTS (16:3/16:3), LPC (20:3), LPC (22:6), LPE (22:6), and PC (18:5e/4:0). The combination of these five differential lipids can be used to distinguish the EOPE from the NC group, with an AUC of 100%, and the LOPE from the NC groups, with an AUC of 99.2%. The ability of the combination to distinguish between the EOPE and the LOPE groups has a diagnostic accuracy of 70.6%. These findings indicate that lipid metabolites have the potential to serve as biomarkers in patients with EOPE or LOPE. Moreover, lipid metabolites correlate with clinical severity indicators for patients with EOPE and LOPE, including fetal birth weight and maternal urine protein levels.

This is the first study of serum untargeted lipidomics changes in EOPE and LOPE. However, our study has certain limitations. The main limitation of our study is that the sample size was small. We identified that the five potential biomarkers for EOPE or LOPE diagnosis were not further confirmed in the dependent validation cohort. Furthermore, due to the underlying pathological mechanism of EOPE, the gestational age of the NC group cannot completely match that of the EOPE group, which may lead to bias. Therefore, based on this study, prospective cohort studies are needed in the future, including expanding the sample size, using different metabolomics detection methods, and fully considering confounding factors such as maternal BMI and gestational age at sampling to deeply explore the role of lipids and other metabolites in the occurrence and development of preeclampsia.

## Conclusion

Our study found differences in lipid metabolism profiles among the normal pregnant women, EOPE, and LOPE. The glycerophospholipid metabolism is the main abnormal serum lipid metabolism pathway between the EOPE and the LOPE. Specific differential lipids were significantly associated with clinical features of EOPE and LOPE, such as maternal urine protein levels and fetal birth weight. Furthermore, we identified five lipids expressed differently among the NC, EOPE, and LOPE, and their combination panel shows great potential for diagnosing EOPE and LOPE. This suggests that lipid metabolites have the potential to serve as biomarkers in patients with EOPE or LOPE. Furthermore, lipid metabolites correlate with clinical severity indicators for patients with EOPE and LOPE, including fetal birth weight and maternal urine protein levels.

### Electronic supplementary material

Below is the link to the electronic supplementary material.

Supplementary Material 1

Supplementary Material 2

Supplementary Material 3

## Data Availability

No datasets were generated or analysed during the current study.

## Abbreviations

Acar: Acylcarnitine
ALT: Alanine aminotransferase
ANOVA: Analysis of variance
AST: Aspartate aminotransferase
AUC: Area under the curve
BMI: Body mass index
BNP: B-type natriuretic peptide
Cer: Ceramide
DAG: Diacylglycerol
DBP: Diastolic blood pressure
EOPE: Early-onset preeclampsia
FAs: Fatty acids
FBW: Fetal birth weight
FC: Fold change
FIGO: Federation international of gynecology and obstetrics
GA: Gestational age
GLs: Glycerolipids
GPs: Glycerophospholipids
IVF: In vitro fertilization
LC-MS: Liquid chromatography-mass spectrometry
LOPE: Late-onset preeclampsia
LPC: Lysophosphatidylcholine
LPE: Lysophosphatidylethanolamine
NC: Normal pregnant controls
OPLS-DA: Orthogonal partial least squares discriminant analysis
PC: Phosphatidylcholine
PCA: Principal component analysis
PE: Phosphatidylethanolamine
PlGF: Placental growth factor
PLT: Platelet
PS: Phosphatidylserine
QC: Quality control
ROC: Receiver operating characteristic
SBP: Systolic blood pressure
sFlt-1: soluble fms like tyrosine kinase-1
SHexCer: Sulfatides hexosyl ceramide
SLs: Saccharolipids
SPs: Sphingolipids
TAG: Triacylglycerol
TIC: Total ion chromatogram
UHPLC-MS/MS: Ultrahigh-performance liquid chromatography-tandem mass spectrometry
VIP: Variable importance in project
WGCNA: Weighted gene co-expression network analysis
