# Supplementary material for: Lipidomic signatures in patients with early-onset and late-onset Preeclampsia
Source: Metabolomics. 2024 Jun 16;20(4):65. doi: 10.1007/s11306-024-02134-x (PMC11180640; doi:10.1007/s11306-024-02134-x)
Supplement: Supplementary file 1 — Supplementary Material 1 [file 11306_2024_2134_MOESM1_ESM.docx]

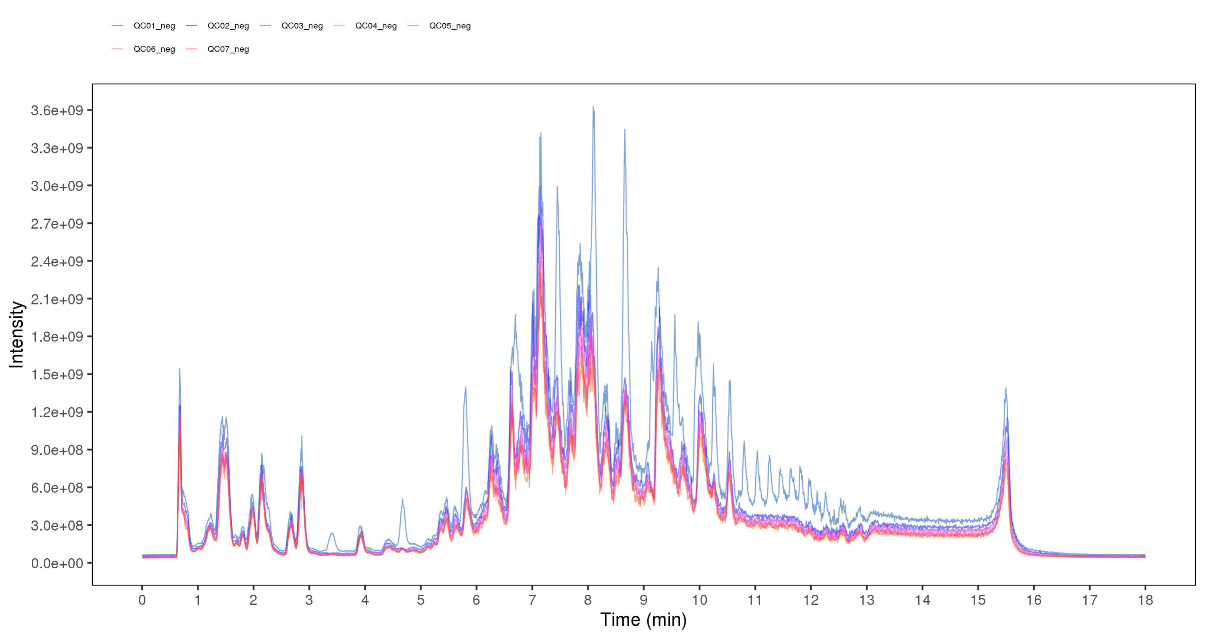


**a**


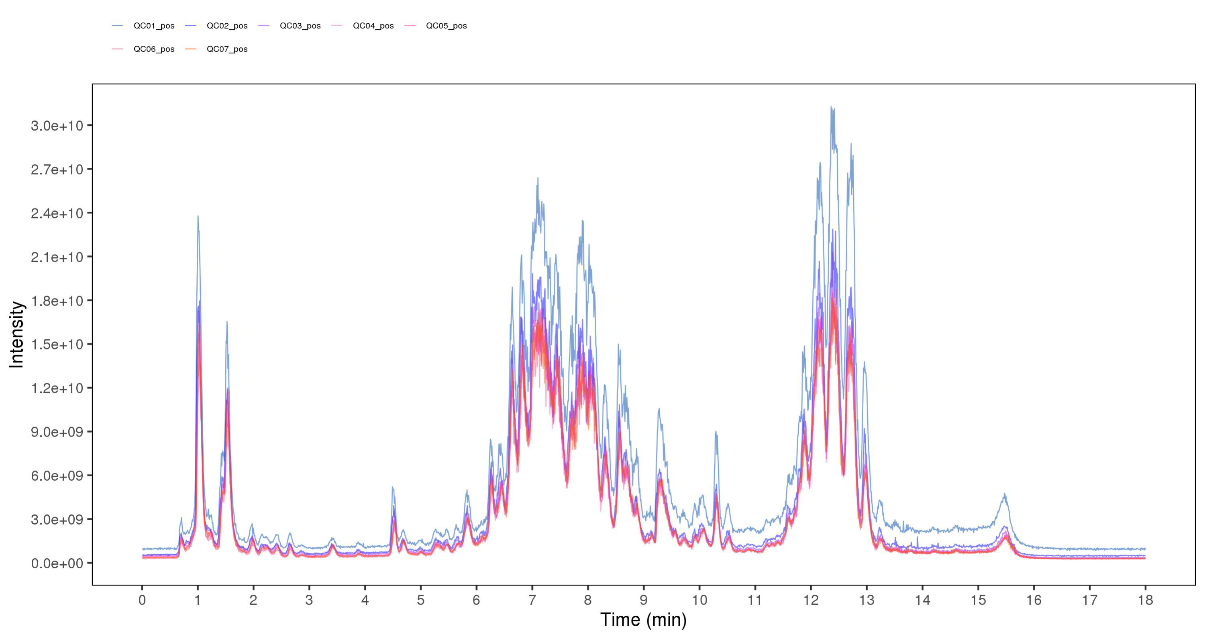


**b**

**Fig. S1** TIC of QC samples. The abscissa represents the retention time of lipid metabolites detection, and the ordinate represents the ion intensity of detection. (a) Total ionization chromatography (TIC) of QC samples in positive ion mode. (b) Total ionization chromatography (TIC) of QC samples in negative ion mode.





**a**


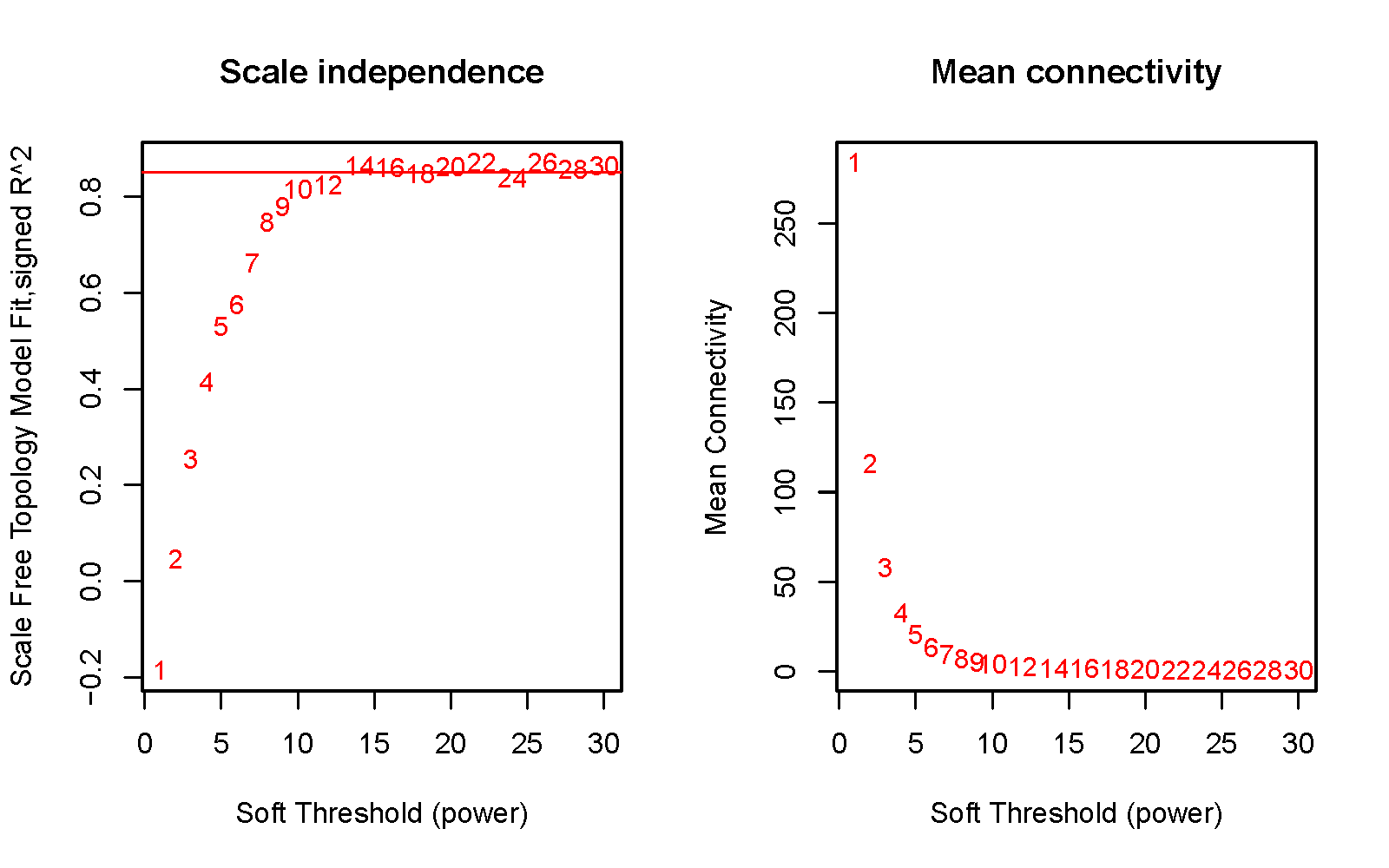


**b**


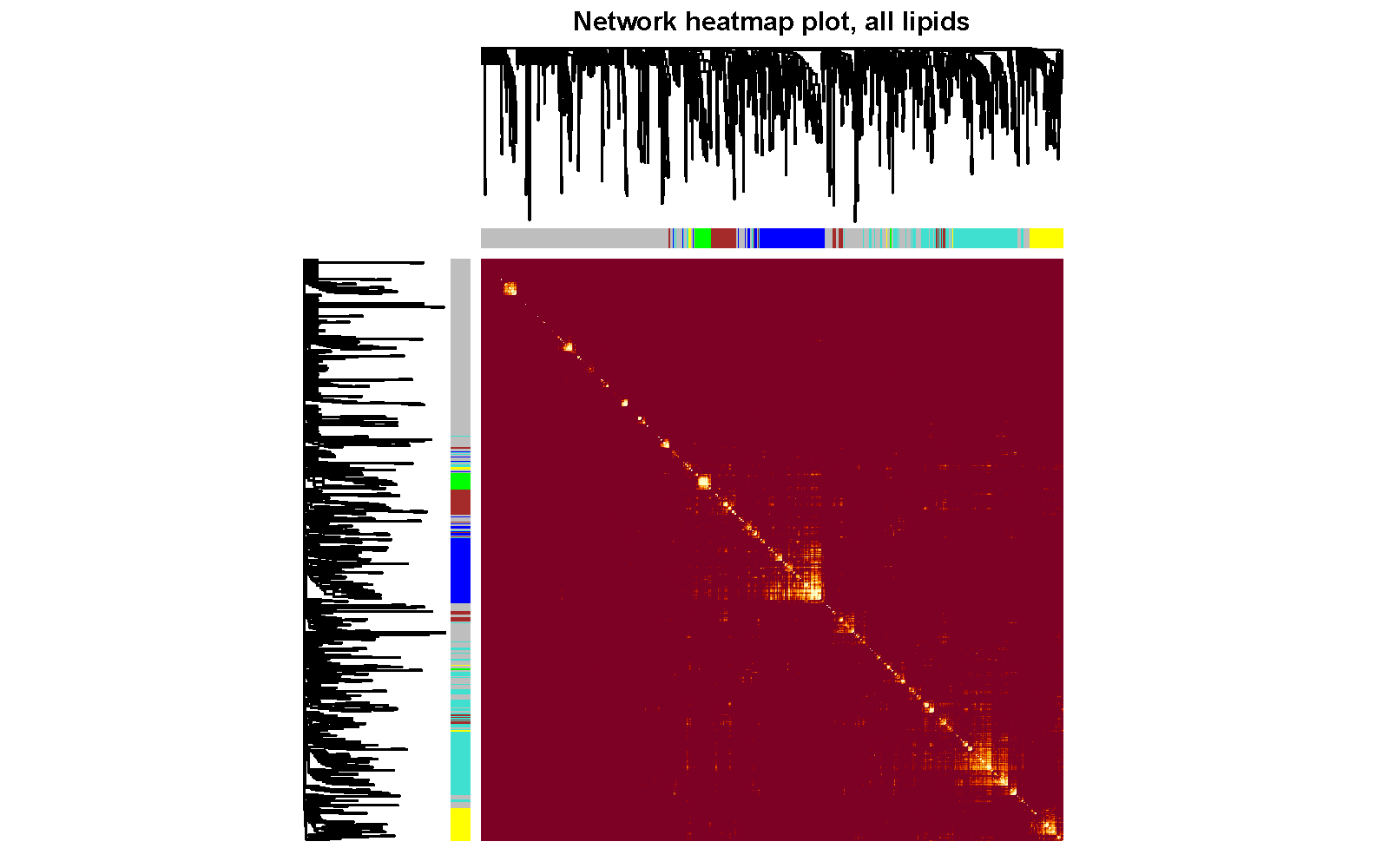


**c**

**Fig.S3** WGCNA analysis to construct the co-expression network among all identified lipids. (a) Hierarchical clustering algorithm to detect outliers. (b) Select the optimal soft-threshold power. (c) Construct the topological overlap matrix (TOM).


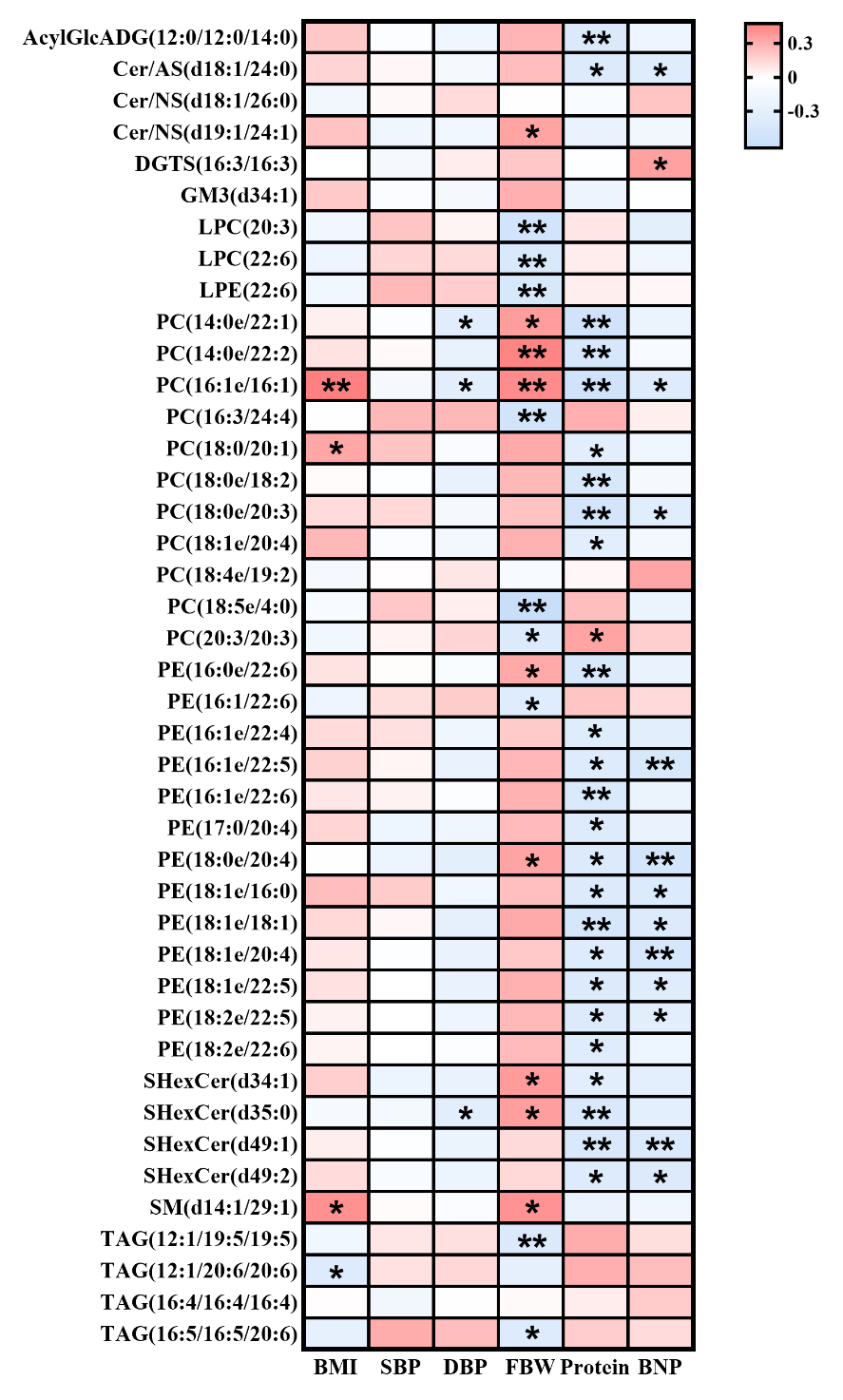


**Fig.S3.** Relationship between maternal serum lipids and preeclampsia clinical parameters. Maternal serum lipids were significantly related to BMI, blood pressure, urine protein (Protein), and fetal birth weight (FBW) in preeclampsia patients. Red denotes a positive connection, blue denotes a negative correlation, and “*” indicates a statistical significance.
